# Transcriptome and biomineralization responses of the pearl oyster *Pinctada fucata* to elevated CO_2_ and temperature

**DOI:** 10.1038/srep18943

**Published:** 2016-01-06

**Authors:** Shiguo Li, Chuang Liu, Jingliang Huang, Yangjia Liu, Shuwen Zhang, Guilan Zheng, Liping Xie, Rongqing Zhang

**Affiliations:** 1 Institute of Marine Biotechnology, Collaborative Innovation Center of Deep Sea Biology, School of Life Sciences, Tsinghua University, Beijing 100084, China; 2 Tsinghua-Peking Joint Center for Life Sciences, School of Life Sciences, Tsinghua University, Beijing 100084, China

## Abstract

Ocean acidification and global warming have been shown to significantly affect the physiological performances of marine calcifiers; however, the underlying mechanisms remain poorly understood. In this study, the transcriptome and biomineralization responses of *Pinctada fucata* to elevated CO_2_ (pH 7.8 and pH 7.5) and temperature (25 °C and 31 °C) are investigated. Increases in CO_2_ and temperature induced significant changes in gene expression, alkaline phosphatase activity, net calcification rates and relative calcium content, whereas no changes are observed in the shell ultrastructure. “Ion and acid-base regulation” related genes and “amino acid metabolism” pathway respond to the elevated CO_2_ (pH 7.8), suggesting that *P. fucata* implements a compensatory acid-base mechanism to mitigate the effects of low pH. Additionally, “anti-oxidation”-related genes and “Toll-like receptor signaling”, “arachidonic acid metabolism”, “lysosome” and “other glycan degradation” pathways exhibited responses to elevated temperature (25 °C and 31 °C), suggesting that *P. fucata* utilizes anti-oxidative and lysosome strategies to alleviate the effects of temperature stress. These responses are energy-consuming processes, which can lead to a decrease in biomineralization capacity. This study therefore is important for understanding the mechanisms by which pearl oysters respond to changing environments and predicting the effects of global climate change on pearl aquaculture.

Ocean acidification (OA) and ocean warming are direct consequences of the increasing carbon dioxide (CO_2_) levels and increasing surface temperatures of the world’s oceans^1^. Increases in CO_2_ levels alter the seawater carbonate system, including dissolved inorganic carbon (DIC, the sum of the solution concentrations of CO_2_ plus carbonic acid [H_2_CO_3_], bicarbonate ions [HCO_3_^–^], and carbonate ions [CO_3_^2–^]), resulting in changes in seawater pH levels. Since the industrial revolution, the mean pH level of seawater has declined by an average of 0.1 units owing to uptake of CO_2_, and the ocean system is predicted to experience a further decrease in pH of 0.3–0.5 units by 2100 and 0.7 units by 2300. Concomitantly, the average seawater surface temperature is predicted to increase by 1.8–6.4 °C by 2100, with a further increase of 2–3 °C by 2300^2^. These predicted changes will likely have adverse effects on the function, structure and fitness of marine ecosystems^1^,^3^,^4^. Indeed, seasonal changes in seawater CO_2_ concentration and temperature induced by the upwelling of CO_2_-enriched seawater or extreme climate events enhance the effects of ocean acidification and warming on marine organisms^5^,^6^.

As the dominant organisms in marine ecosystems, marine calcifiers are vulnerable to OA because of their weak acid-base regulation ability, which especially affects the biomineralization process^7^; in these species, biomineralization (also termed biocalcification) is the key physiological process for producing calcium carbonate (CaCO_3_). It has been shown that OA can lead to decalcification of the shell and skeleton of marine calcifiers^8^,^9^, and ocean warming also has marked effects on biocalcification^10^. Importantly, the impacts of these stressors on biomineralization have significant functional and ecological implications because of the defensive functions of calcified tissues in marine ecosystems.

The pearl oyster *Pinctada fucata* (also named *P. martensii*), an economically and ecologically important marine calcifier, is one of the best studied species with respect to biomineralization processes during pearl and shell formation^11^,^12^. Along the coast of the South China Sea (SCS), the pH and temperature of the surface seawater fluctuate from a minimum of 8.1 and 19 °C in winter to a maximum of 7.6 and 30 °C in summer^13^,^14^, and the large fluctuations in seawater pH and temperature in this area impose demands on the ability of pearl oysters to adapt to these changes^15^. Although wide-ranging effects of OA and ocean warming on the physiological performances of marine calcifiers, including pearl oysters, have previously been demonstrated^10^,^16^,^17^,^18^,^19^,^20^, it remains unclear how pearl oysters can adapt to large-scale, rapid changes in pH and temperature that are likely to be associated with near-future ocean acidification and warming. Thus, there is a critical need for a comprehensive understanding of the strategies used by pearl oysters to adapt to these changes. Transcriptome analysis is an excellent approach for investigating the responses of marine organisms to environmental changes^21^, and abundant transcriptome information about *P. fucata*, which can be used in our study, has been reported^22^,^23^ in recent years.

The aims of this study were to elucidate the potential molecular/cellular mechanisms underlying the physiological responses of *P. fucata* to rapid temperature and pH stress and to examine the effects of these stressors on biomineralization. A microarray-based approach was used to investigate the transcriptome responses of *P. fucata* to elevated CO_2_ and temperature, and the effects of these stressors on biomineralization were analyzed by measuring the alkaline phosphatase activity, the net calcification rate and the calcium content and by observing the shell ultrastructure. The mantle was selected as the test sample because it is the organ responsible for sensory functions, accessory respiration and biomineralization in pearl oysters^24^, organisms that have the ability to discriminate among and defend against unfavorable seawater environments. Because this study focuses on the responses of *P. fucata* to changing seawater environments, the imposed stressors mimic the fluctuations of pH and temperature in the SCS (pH 7.8, pH 7.5, 25 °C and 31 °C), such that the levels are equivalent to the predictions for OA and warming for the years 2100 and 2300.

## Results

### Biomineralization of the shell

**Figure 1:**
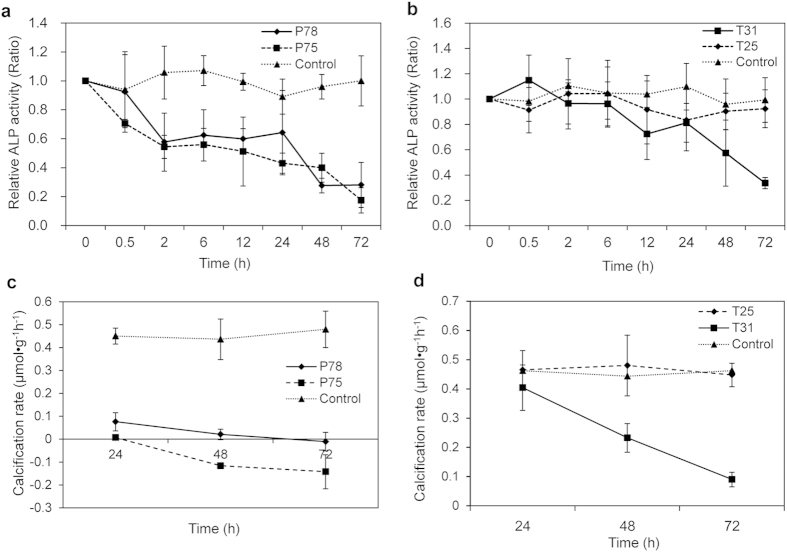
The relative activity of mantle alkaline phosphatase (ALP, (a,b)) and net calcification rate (NCR, (c,d)) of *Pinctada fucata* under CO_2_ and temperature stress. The ALP and NCR data are presented as the ratios between the treatment group and the control at the corresponding time point.

In Fig. 1a,b, the alkaline phosphatase (ALP) activity in the control shows no significant difference between the 0 time point and each experimental time point (*p* > 0.05). At the 72 h time point, the ALP activities decrease by 71.94% in P78 and 82.53% in P75 (from 17.14 nmol pNP•min^−1^•mg^−1^ to 4.8 nmol pNP•min^−1^•mg^−1^ and from 17.14 nmol pNP•min^−1^•mg^−1^ to 2.91 nmol pNP•min^−1^•mg^−1^, respectively) compared with that of the control (*p* < 0.05). Although the temperature stress in T25 at 48 h and 72 h does not affect the ALP activities compared with the control (from 16.58 nmol pNP•min^−1^•mg^−1^ to 15.63 nmol pNP•min^−1^•mg^−1^ and from 17.24 nmol pNP•min^−1^•mg^−1^ to 16.04 nmol pNP•min^−1^•mg^−1^, respectively), the ALP activity decreases by 42.66% at 48 h and 66.31% at 72 h in T31 (from 16.58 nmol pNP•min^−1^•mg^−1^ to 9.45 nmol pNP•min^−1^•mg^−1^ and from 17.24 nmol pNP•min^−1^•mg^−1^ to 5.86 nmol pNP•min^−1^•mg^−1^, respectively, *p* < 0.05).

The net calcification rate (NCR) in P78 reduces by 83.12% at 24 h, 95.26% at 48 h and 102.28% at 72 h compared with the control at the corresponding time points. For P75, the NCR decreases by 85.07% at 24 h, 126.67% at 48 h and 129.71% at 72 h (*p* < 0.05, Fig. 1c). As shown in Fig. 1d, temperature stress in T25 causes the NCR to increase by 0.64% at 24 h and 8.28% at 48 h and decrease by 3.13% at 72 h compared with the control at the corresponding time points (*p* < 0.05). Additionally, the NCR reduces by 12.54% at 24 h, 47.54% at 48 h and 80.54% at 72 h, in T31 compared with the control at the corresponding time points (*p* < 0.05).

**Figure 2:**
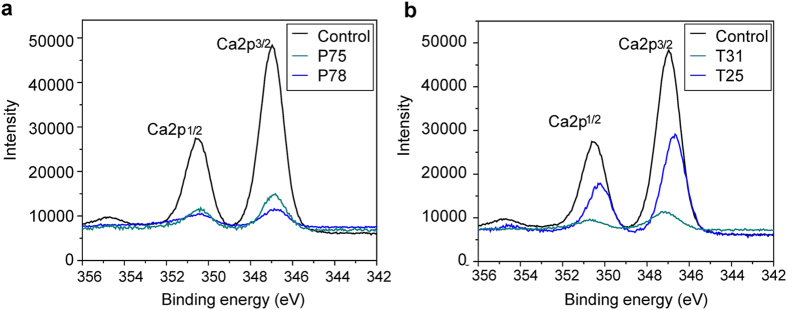
The relative calcium content on the inner surface of the shell near the nacre-prism transition region in *Pinctada fucata* under CO_2_ (a) and temperature (b) stress.

Reduced pH (pH 7.8 in P78 and pH 7.5 in P75) has a significant negative effect on the relative calcium content on the surface of the shell (Fig. 2a). Similarly, the relative calcium content decreases with an increase in temperature from 19 °C to 25 °C in T25 and from 19 °C to 31 °C in T31 (Fig. 2b).

Normal stair-like growth patterns of aragonite on the nacreous layer and normal prism-like growth patterns of calcite on the prismatic layer are observed in the shells in the control group. With growth patterns similar to the control, CO_2_ and temperature do not result in significant changes in shell ultrastructure (Supplementary Fig. 1).

### Amino acid contents of the mantle tissue

In the mantle tissue, the exposure to pH7.8 leads to a significant increase in Phe and decrease in Gly content compared with the control (*p* < 0.05; Supplementary Table 1). Phe, Cys, Met and Ala are significantly increased in P75 at pH 7.5, whereas Tyr, Glu and Gly are decreased after this treatment compared with the control (*p* < 0.05). In contrast, exposure to 25 °C in T25 and 31 °C in T31 do not induce significant changes in amino acid content, and no significant changes in Arg, Asn and Gln after any of the treatments are found compared with the control.

### Transcriptomic analysis of the mantle tissue

A total of 5862, 6555, 3181 and 3730 differentially expressed unigenes (DEGs, *p* < 0.05 and change fold > 2) for P78, P75, T25 and T31 are identified from a microarray, accounting for 10.01%, 11.19%, 5.43% and 6.49% of the total genes, respectively (Supplementary Fig. 2a,b). Clustering analysis for the DEGs shows excellent repeatability and characteristic gene expression profiles for the different treatments (Supplementary Fig. 2c). The expression data obtained by microarray and real-time quantitative PCR (RT-qPCR) exhibited the same trend, with similar expression levels. Highly significant correlations (R^2^ = 0.9429 in P78, 0.8885 in P75, 0.9449 in T25 and 0.8399 T31) between the microarray and RT-qPCR results are obtained, confirming the reliability of the microarray results (Supplementary Table 2 and Supplementary Fig. 3).

**Figure 3:**
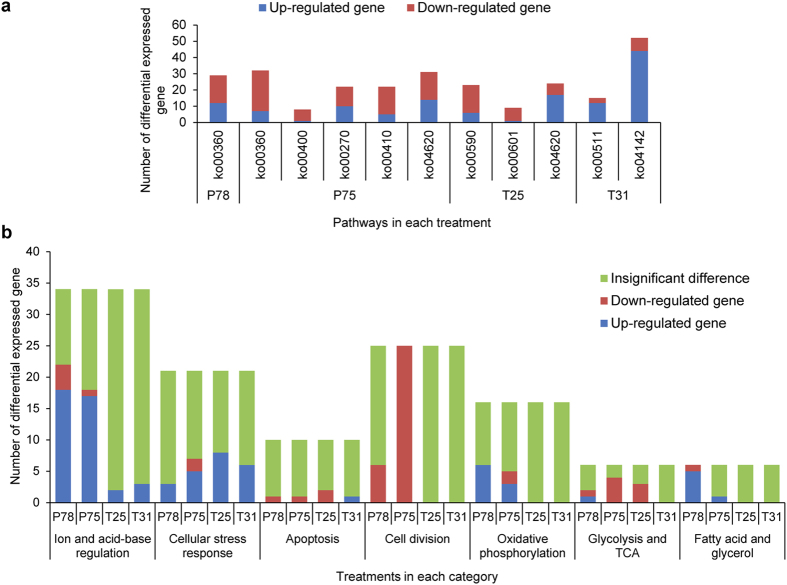
The pathway (a) and category (b) enrichments of differentially expressed genes (DEGs) in *Pinctada fucata* exposed to CO_2_ and temperature stress. (**a**) ko00360: Phenylalanine metabolism; ko00400: Phenylalanine, tyrosine and tryptophan biosynthesis; ko00270: Cysteine and methionine metabolism; ko00410: beta-Alanine metabolism; ko00590: Arachidonic acid metabolism; ko00601: Glycosphingolipid biosynthesis; ko00511: Other glycan degradation; ko04142: Lysosome; ko04620: Toll-like receptor signaling pathway.

Enrichment analyses indicate 29, 115, 56 and 67 DEGs are assigned to 1, 5, 3 and 2 over-represented pathways (*p* < 0.05) in P78, P75, T25 and T31, respectively (Fig. 3a, Supplementary Table 3). It is noteworthy that a total of 58.62% and 72.62% of the unigenes involved in the category “amino acid metabolism” are down-regulated in P78 and P75, respectively, suggesting a significant metabolic depression. The most representative pathway in P78 and P75 is “phenylalanine metabolism”. The “Toll-like receptor signaling pathway” in T25 and the “lysosome” and “other glycan degradation” pathways in T31 are activated, as assessed by unigene up-regulation, whereas the other pathways are suppressed by the four treatments (Supplementary Table 3).

The unigenes involved in the categories “ion and acid-base regulation”, “cell stress responses”, “apoptosis” and “cell division” are highlighted in this study (Fig. 3b, Supplementary Table 4). With regard to ion and acid-base regulation, few gene expression alterations are observed after the temperature treatments, whereas the expression levels of these genes are significantly up-regulated after the CO_2_ treatments, particularly for vacuolar type H^+^-ATPase (V-ATPase), sodium/potassium-transporting ATPase (NKA), sodium/hydrogen exchanger (NHE3/5), sodium/potassium/calcium exchanger (NCKX), anion/bicarbonate transporter family members (SLC26A3/4/6), chloride channel (CLC), voltage-dependent calcium channel (VDCC) and sarco-endoplasmic reticulum calcium transport ATPase (SERCA). The expression levels of a sodium bicarbonate cotransporter (NBC1/3) and anion exchanger (AE2) do not show significant differences. For the category of “cellular stress response”, the expression levels of heat shock protein 70 (HSP70), which is involved in the heat shock response, are up-regulated in response to the four treatments. In addition, the unigenes involved in “oxidation and anti-oxidation” are also up-regulated in response to increased temperature: these included cytochrome P450 (CYP) in T25 and T31, and glutathione S-transferase (GST) and glutathione peroxidase (GPO) in T25. The gene expression patterns indicate that cell apoptosis might occur in T31. The “cell division”-related unigenes are unchanged in T25 and T31, although down-regulation is found in P78 and P75.

Unigenes [F0F1-type ATPase (F-ATPase), adenylate cyclase (AC), complex I (CI) and protein kinase A (PKA)] involved in “oxidative phosphorylation” (OP) are up-regulated in P78, but not in P75, suggesting a significant increase in ATP production (Supplementary Table 5). The expression levels of unigenes involved in “glycolysis” and the “tricarboxylic acid cycle” (TCA) indicate that glycolysis is suppressed and the TCA is activated in P78. Furthermore, unigenes involved in “fatty acid metabolism”, but not “glycerol metabolism”, are up-regulated in P78, suggesting accelerated fatty acid degradation.

**Figure 4:**
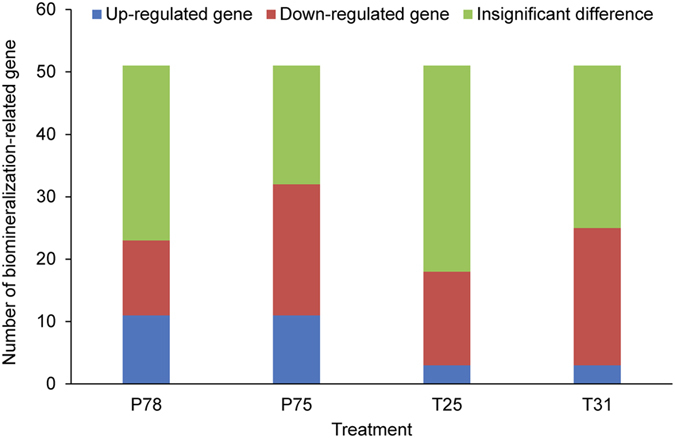
Expression changes in biomineralization-related genes in *Pinctada fucata* exposed to CO_2_ and temperature stress.

To understand biomineralization responses, changes in 51 biomineralization-related unigenes from the microarray are analyzed separately (Fig. 4, Supplementary Table 6): 80.39% are differentially expressed in response to at least one treatment. Down-regulation of the unigenes encoding tyrosinase (TYR) in P75, T25 and T31 and up-regulation of tyrosinase-like proteins (TYRL1 and TYRL2) in P78 and P75 are observed. In addition, the expression levels of unigenes encoding chitin synthase (CHS), nacrein and carbonic anhydrase precursor (CA) are down-regulated, although CA12 isoform is not affected.

## Discussion

**Figure 5:**
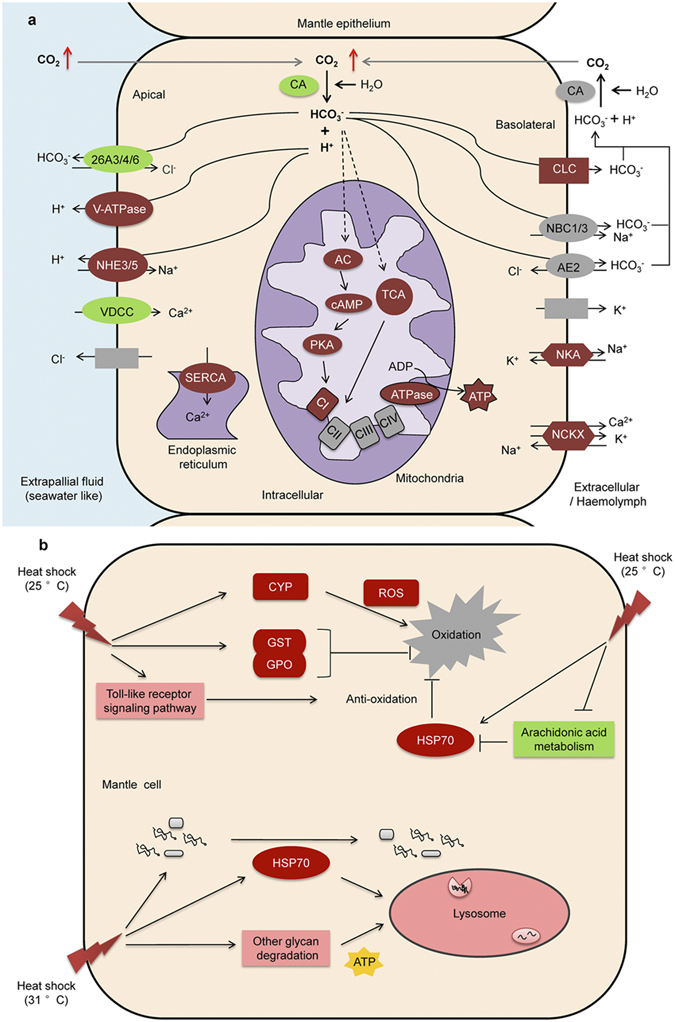
Schematic representations of the mechanisms underlying the response of mantle cells of *Pinctada fucata* to CO_2_ and temperature stress. (**a**) Acid-base regulatory strategy in *P. fucata* is involved in the response to elevated CO_2_. The homeostasis of intracellular H^+^ and HCO_3_^−^ are regulated by cytoplasmic carbonic anhydrase (CA), membrane-associated CA12, and apical and basolateral proton pumps, ion channels, exchangers and transporters, including increased expression of vacuolar type H^+^ -ATPases (V-ATPase), sodium/hydrogen exchangers (NHE3/5), chloride channels (CLC), sodium/potassium-transporting ATPases (NKA) and sodium/potassium/calcium exchangers (NCKX), decreased expression of anion/bicarbonate transporter family members (SLC26A3/4/6), and normal expression of sodium bicarbonate cotransporters (NBC1/3), anion exchangers (AE2), and K^+^ channels. In these processes, increased energy demand is supplemented by enhancement of the tricarboxylic acid cycle (TCA) and oxidative phosphorylation (OP) is stimulated by HCO_3_^−^ *via* adenylate cyclase (AC)-induced signaling and membrane transport, including increased expression of complex I (CI), F0F1-type ATPases (F-ATPase), AC, cAMP and protein kinase A (PKA). In the diagram, crimson indicates up-regulation, green indicates down-regulation, and gray indicates insignificant changes. The solid arrows show the direction of ions and CO_2_ diffusion. The dotted arrows show the hypothetical signaling pathways. (**b**) Anti-oxidative and lysosome pathways in *P. fucata* are involved in the response to elevated temperature. Elevated temperature induces the production of reactive oxygen species (ROS) *via* increased expression of cytochrome P450 (CYP), resulting in oxidative stress. Under the medium temperature stress (25 °C), the increased expression of “anti-oxidation”-related genes [e.g., heat shock protein 70 (HSP70), glutathione S-transferase (GST) and glutathione peroxidase (GPO)], activation of the “Toll-like receptor signaling pathway” and depressed “arachidonic acid metabolism” promote anti-oxidative regulation in mantle cells. Under high temperature stress (31 °C), the “lysosome” pathway is the main defense system for clearing damaged proteins and organelles, with assistance from HSP70. The energy supplement might originate from the enhanced degradation of other glycans. In this diagram, crimson indicates up-regulated genes, green indicates down-regulated genes, pink indicates enhanced pathways, blue indicates inhibited pathways, and gray indicates oxidative stress.

By comparing changes in the expression levels of genes involved in the categories “ion and acid-base regulation”, “cell stress responses”, “apoptosis” and “cell division”, the regulation of genes involved in “ion and acid-base regulation” emerges as the principal transcriptome response of adult *P. fucata* to CO_2_ stress. This finding is consistent with findings in sea urchins but not in corals^25^,^26^. Therefore, we hypothesize the specific mechanism involved in this response (Fig. 5a). Based on the results of functional studies in marine organisms and vertebrates, we propose that the homologs of genes involved in the response perform similar functions in the *P. fucata* mantle.

Carbonic anhydrases, which catalyzes CO_2_ and HCO_3_^−^ interconversion, appears to be sensitive to OA in marine calcifiers^27^. Indeed, the down-regulation of cytoplasmic CA and normal expression of membrane-associated CA12 in *P. fucata* are proposed to be responsible for preventing an excessive increase in intracellular HCO_3_^−^ and H^+^, and the down-regulation of apical SLC26As is responsible for impeding the outflow of HCO_3_^−^ to seawater^28^. Thus, redundant H^+^ might be excreted *via* apical V-ATPase or NHE3/5^29^. HCO_3_^−^ is enriched in the interstitial fluid or hemolymph through basolateral CLCs, which are predicted to be located in the basolateral membrane of epithelial cells in crab gills and function in osmoregulation and acid-base regulation^30^. Moreover, significant permeability of CLC-5 and CLC-Kb to HCO_3_^−^ has been demonstrated^31^. These data support the predictive function of CLC in the mantle epithelium. Similar to the dissolution of shells under seawater acidification^32^, the accumulation of HCO_3_^−^ can neutralize the hypercapnia induced by elevated CO_2_. Increased NKA and NCKX abundance provides transmembrane gradients of Na^+^ and K^+^ for the exchangers, and Cl^−^ and K^+^ recycling might occur transcellularly *via* apical Cl^−^ and basolateral K^+^ channels. In addition, as described for crabs and sharks^30^,^33^, an AC-induced signaling pathway is proposed to stimulate the OP process, based on the up-regulation of AC, cAMP, PKA and CI, which trigger the supplemental energy required for acid-base regulation. However, unlike fish^34^, such energy supplementation might be fuelled by the breakdown of fatty acids but not amino acids or carbohydrates. Our data therefore do not support the hypothesis that metabolic depression is accompanied by suppression of ATP generation^25^ yet support the idea that metabolic depression occurs through the active suppression of metabolism.

In our model, *P. fucata* is able to compensate for the acid-base homeostasis disturbance caused by reduced pH at the level predicted for the year 2100 (~pH 7.8) *via* transmembrane movement of H^+^ and HCO_3_^−^. However, this regulation is not possible at ~pH 7.5, which is predicted for the year 2300, owing to insufficient compensation ability, suggesting that such stress will exceed the resistance thresholds of crucial physiological processes. The result indicates that such conditions might cause significant metabolic depression in pearl oysters, which is confirmed by the down-regulation of metabolism-related genes. In addition, the Phe, Cys, Met and Ala content is increased in the mantle of *P. fucata* exposed to higher CO_2_ levels, which is indicative of the metabolic depression of these amino acids. Overall, the changes in amino acid content support the gene expression results. Furthermore, physiological data on changes in metabolic rates in this species after exposure to acidified seawater support our conclusion^35^. In marine calcifiers, metabolic depression is considered to be a short-term strategy that allows the organism to tolerate seawater acidification^36^,^37^, by decreasing energy costs and metabolic CO_2_. By reducing amino acid metabolism, *P. fucata* maintains enough energy to satisfy its physiological needs, such as ion and acid-base homeostasis. Owing to the energy trade-off, the compensation for seawater acidification will affect the physiological performance of the oyster, including mantle cell division. Given that the mantle is an important organ responsible for predation, defense and biomineralization, these effects may have ecological and functional consequences for pearl oysters if they are tested over a longer period of time.

Previous studies have shown that *P. fucata* is resistant to heat stress^20^,^38^. The underlying strategy involved in this tolerance can be summarized from transcriptome changes. In this study, the expression of stress response-related genes is significantly affected by increased temperature. According to our results, and in contrast to *C. virginica*, in which stress levels play a relatively small role^39^, we hypothesize that the *P. fucata* response to temperature stress depends on the extent of the temperature rise (Fig. 5b).

Ocean warming is predicted to affect marine ecosystems at different levels of biological organization. Elevated temperature can enhance oxygen consumption in aquatic animals and therefore may rapidly produce reactive oxygen species (ROS), resulting in oxidative stress^40^. For a medium-level temperature (25 °C), the anti-oxidative system is used by pearl oysters to address oxidative damage; this is also the mechanism employed by the blue mussels *Mytilus trossulus* and *M. galloprovincialis* and the Pacific oyster *C. gigas*^41^,^42^, with genomic analyses revealing that the specific induction of HSP70 and its high level of expression are critical for *C. gigas* to adapt to highly stressful environments^42^. Similarly, HSP70 in *P. fucata* may play a central role in coping with temperature stress by eliminating the effects of ROS production^43^. This defense is further assisted by the up-regulation of antioxidant genes, such as GST and GPO^42^. The “arachidonic acid metabolism” pathway might also assist in the responses, suggesting potential feedback regulation for HSP70^44^. Furthermore, consistent with pathways found for *Chlamys farreri*^45^, it is reasonable to suggest that stimulation of the TLR pathway might increase the anti-oxidative capacity of *P. fucata*. Moreover, in the T31 condition of the current study, the “lysosome” pathway was initiated to reduce oxidative stress. With the enhancement of apoptosis capacity in mantle cells, HSP70 may have acted as a molecular chaperone that promotes the transport of proteins toward lysosomes^46^ or that stimulates lysosomal catabolism^47^ to clear away the damaged proteins and organelles produced by oxidative stress. The additional energy for this regulation might derive from the enhanced degradation of glycans. Through such regulation, pearl oysters can sustain mantle cell performance (e.g., cell division). Overall, the results indicate that *P. fucata* has an enhanced capacity to ameliorate temperature stress.

In fact, the daily and seasonal changes in pH and temperature in the natural seawater that pearl oysters inhabit are similar to what is predicted for the modifications in the near-further ocean. According to the results of short-term experiments, it could be proposed that *P. fucata* can resist the stress caused by rapid seawater changes by regulating its physiological status.

The transcriptome analysis in the present study also indicates that *P. fucata* biomineralization is sensitive to changes in pH and temperature. As biomineralization is thought to be an energetically costly process in marine invertebrates, the regulation implemented in response to increased CO_2_ and temperature is likely to suppress biomineralization by affecting energy-requiring transport steps^26^,^37^. During the biomineralization process, it has been hypothesized that inorganic carbon and calcium are first concentrated in mantle cells, producing amorphous calcium carbonate (ACC), and then enter specialized vesicles before being transported from the mantle to the biomineralization site *via* exocytosis^48^. Down-regulation of apical VDCC^49^, up-regulation of SERCA in the ER^49^ and up-regulation of basolateral NCKX^50^ lead to a decrease in the net calcium content of mantle cells, directly inhibiting ACC production or indirectly affecting biomineralization *via* calcium-dependent signaling pathways. From a physiological perspective, calcium absorption and ACC formation are related to ALP, a hydrolase enzyme that is affected by heat stress^51^ and is regarded to be a marker for biomineralization activity in mollusks^52^. The expression level of ALP and its activity are both down-regulated under exposure to elevated CO_2_ and temperature. Such trends are also found for CA and calcium binding-related genes. The significant consequence of these changes is the down-regulation of NCR, which is strongly responsive to increased CO_2_ and temperature in marine calcifiers^21^.

Studies on the responses of biomineralization-related genes to OA and ocean warming have found diverse expression patterns^20^,^26^,^53^, and these observations are corroborated in the current study. Although most of these genes are differentially expressed in response to increases in CO_2_ and temperature, individuals respond differently, suggesting a complex relationship between gene expression and stressors. The genes encoding tyrosinase and chitin synthase are highlighted here, as they show larger fold changes in the microarray experiments than did other responsive genes. Tyrosinase plays an important role in the formation of the prismatic layer and the periostracum, which is a protective layer in the shells of pearl oysters. The function-specific response hypothesis about *M. edulis*^54^ might aid in understanding our results: TYR is responsible for melanogenesis, which is negatively regulated by CO_2_ and temperature stresses; conversely, increased expression of TYRL1 and TYRL2 enhance the synthesis of tyrosinase, strengthening the periostracum and prismatic layer to resist stress. In mollusk shells, chitin is proposed to form the matrix structure on which matrix proteins can control CaCO_3_ crystal growth^55^. In contrast to *M. edulis* and *Laternula elliptica*^54^,^56^, the expression of CHS in *P. fucata* exposed to acidified seawater and temperature stress (high levels) was down-regulated, which might lead to serious effects on frame organization during biomineralization.

In *P. fucata*, phenylalanine metabolism was enriched during exposure to increased CO_2_ concentrations. Phenylalanine, a biologically essential amino acid, generates metabolic products comprising a range of different substances, including phenethylamine. Interestingly, phenethylamine degradation has been considered to depress the expression of calcification-related genes in *S. purpuratus* exposed to OA^27^. Therefore, the depression of phenylalanine metabolism in *P. fucata* might inhibit its biomineralization ability by influencing the expression of related genes that might be regulated by phenethylamine.

Combined with changes in biomineralization ability and ion status, changes in the CO_2_ levels and temperature will have a negative influence on crystal growth. Although the calcium content on the surface of the shell and the NCR decreased, SEM showed that the ultrastructure of aragonite and calcite on the nacreous and prismatic layers was not affected. Previous investigation has indicated that biomineralization is a complex process, and the effects of CO_2_ levels and temperature depend upon the duration of exposure^42^. Biomineralization is also controlled by various other factors, such as ions, environmental factors and various genes. In our study, the effects of CO_2_ and temperature stresses were not manifested in the shell ultrastructure, although the process of biomineralization was affected. Importantly, the nacreous layer of pearl oyster shells is structurally similar to pearls, and the impact of environmental stressors on pearls can actually be reflected in the shells. If the stressors are long-term events, they will have adverse consequences on the biomineralization of pearls, most likely affecting pearl quality, resulting in substantial economic losses for the aquaculture industry. Our study therefore might have potential implications for predictions of the effects of global climate change on pearl aquaculture. Further studies are needed to directly test the physiological and biochemical changes that are suggested by our gene expression data (e.g., the physiology of ion transporters), as changes in cellular proteins may not absolutely coincide with the abundance of the corresponding mRNAs^57^.

## Conclusion

Transcriptome analysis is a powerful tool for revealing the molecular mechanisms underlying the responses of pearl oysters to environmental stressors. The results show that CO_2_ and temperature stresses induce significant changes in the transcriptome and in biomineralization. There is evidence that *P. fucata* might be able to mitigate the effects of elevated CO_2_ (pH 7.8) by regulating the acid-base equilibrium and ameliorate the effects of temperature stress (25 °C and 31 °C) by regulating anti-oxidants and lysosomes. Clearly, these responses are energy-consuming processes, which have adverse effects on biomineralization capacity. This study therefore is important for understanding the mechanisms underlying the responses of pearl oysters to changing seawater environments and also has implications for predicting the effects of global climate change on pearl aquaculture.

## Methods

### Organism collection and experimental design

Adult pearl oysters *Pinctada fucata* used in this study were obtained from the Marine Biology Research Station in Leizhou Bay, China. The seawater conditions of the sample collection area were 19.0 °C, pH 8.1, and salinity 33.0 psu. Prior to use, *P. fucata* was pre-cultured at 19.0 ± 0.5 °C, pH 8.1 ± 0.05 and salinity 33.0 ± 0.5 psu in glass aquaria filled with artificial seawater (Formula Grade A Reef Sea Salt, Formula, Japan). After an acclimation period of two weeks, organisms of similar size (6–7 cm length) were assigned to 50 L tanks and subjected to CO_2_ and temperature stressors. The CO_2_ stress mimicked the seasonal changes in the SCS and the predicted pH levels for the years 2100 (pH 7.8) and 2300 (pH 7.5). The temperature stress mimicked the seasonal changes and temperature levels predicted for 2100 (medium-level at 25 °C) and 2300 (high-level at 31 °C). The *P. fucata* oysters were maintained for 72 h under the following conditions: 19.0 °C, pH 7.8 and salinity 33.0 psu (group denoted P78); 19.0 °C, pH 7.5 and salinity 33.0 psu (P75); 25.0 °C, pH 8.1 and salinity 33.0 psu (T25); 31.0 °C, pH 8.1 and salinity 33.0 psu (T31). *P. fucata* cultured synchronously in 50 L tanks at 19.0 °C, pH 8.1 and salinity 33.0 psu were used as the control. Each treatment was carried out with three replicates in three independent tanks, and each tank contained 30 individuals. The pearl oysters were daily fed *ad libitum* with the microalgae *Platymonas subcordiformis*.

Samples from three individuals randomly selected from each tank were pooled to obtain one biological replicate. For physiological and molecular analyses, mantles in the control and treatment groups were collected at 0, 0.5, 2, 6, 12, 24, 48 and 72 h, washed with sterilized seawater and tested immediately. For shell analyses, the shells in the control and treatment groups were collected at 0, 24, 48 and 72 h, washed with deionized water and immersed in 5% sodium hydroxide for 12 h to remove organic components attached to the inner surface. The shells were then washed thoroughly with deionized water, air-dried and stored in a desiccator until required.

### Seawater chemistry

The pH values were maintained by continuously pumping a CO_2_-gas mixture into the tanks and monitored using a pH-201 digital pH controller (IPA, Shenzhen, China). Temperatures were controlled by inserting a heating rod with a D838-100 digital temperature controller (UP, Taiwan) at the bottom of the tank. pH and temperature values were recorded daily, and they did not deviate from the expected levels during the exposures (Supplementary Table 7). Initial and final seawater samples were collected from each tank, and three replicates were taken to determine total alkalinity (TA) and total dissolved inorganic carbon (DIC). The partial pressure of carbon dioxide (*p*CO_2_) and other parameters were calculated using the DOS-based carbonate chemistry calculation software CO_2_SYS^58^, with the dissociation constants *K*_1_, *K*_2_ and *K*_SO4_^−^.

### ALP activity

ALP activity was measured in 0.1 g of mantle tissue using the colorimetric ALP assay kit (Beyotime Institute of Biotechnology, Haimen, China) following the manufacturer’s instructions. After the addition of p-NitroPhenol Phosphate to each sample and incubation for 10 min at 37 °C, absorbance was measured at 405 nm using an Ultrospec 7000 (GE Healthcare Life Sciences, Uppsala, Sweden). The p-NitroPhenol concentrations used for drawing the standard curve were 0.02, 0.04, 0.08, 0.12 and 0.16 mM. To verify the stability of the control, ALP activities in the control at 0.5, 2, 6, 12, 24, 48 and 72 h were compared with that at 0 h. To eliminate the influence of other factors, ALP activities in P78, P75, T25 and T31 were compared with that of the corresponding control, at 0.5, 2, 6, 12, 24, 48 and 72 h.

### Net calcification rate (NCR)

The TA change in seawater was used to calculate the NCR using the alkalinity anomaly technique, as previously described^59^; this technique is commonly used to calculate calcification rates for marine calcifiers. The NCR values at time points 24, 48 and 72 h are expressed as μmol CaCO_3_ •g^−1^ h^−1^.

### X-ray Photoelectron Spectroscopy (XPS)

X-ray Photoelectron Spectroscopy (XPS, ESCALAB 250Xi, Thermo Scientific, USA) with monochromatic Al Ka radiation (1486.7 eV) was used to analyze the relative calcium content in the nacreous layer on the inner surface of the shell near the nacre-prism transition region. Three shell samples with side lengths of 7 mm × 7 mm were collected from pearl oysters in each treatment and control. To avoid individual error across oysters, three areas at the position along the nacre-prism transition region were tested for each shell sample. The shell samples were irradiated with x-rays with an incident angle of 45^o^. All binding energies were charge-corrected to the adventitious C (1 s) peak at 285 eV, and the measurement precision of the binding energy was 0.2 eV. High-resolution C (1 s) and Ca (2p) spectra were obtained at a pass energy of 50 eV. The relative atomic concentration ratio of the elements on the shell surface was calculated by Advantage V4 (Thermo-VG Scientific, Sussex, England) using the sensitivity factors and signal intensities of the elements.

### Scanning electron microscopy (SEM)

To evaluate whether increased CO_2_ and temperature affects shell ultrastructure, the inner surface of the shell samples (near the nacre-prisms transition region) were cut into small pieces, and sections of the nacreous and prismatic layers were observed by scanning electron microscopy (SEM, FEI Quanta 200, Netherlands), as previously described^19^.

### Liquid chromatography-tandem mass spectrometry (LC-MS/MS)

The amino acid contents in the mantle of *P. fucata* were determined by LC-MS/MS. The amino acid selections were based on the results of the enriched pathways shown in supplementary Table 3: phenylalanine (Phe), cysteine (Cys), Tyrosine (Tyr), methionine (Met) and alanine (Ala). Moreover, glutamic acid (Glu), glycine (Gly), arginine (Arg), asparagine (Asn) and glutamine (Gln) were also used as reference amino acids. The detailed method for LC-MS/MS is provided in Supplementary Information.

### Microarray experiment

A 58,583-feature microarray representing 58,583 unigenes was developed on the basis of the mantle transcriptome of *P. fucata*. The microarray data have been deposited in Gene Expression Omnibus (http://www.ncbi.nlm.nih.gov/geo/; GSE57171) and Dryad (doi:10.5061/dryad.958sr). Detailed methods for the microarray and RT-qPCR validation are provided in Supplementary Information.

DEGs were identified using unpaired Student’s t-tests by comparing the normalized signal values of four treatments (T25, T31, P78 and P75) with that of the control at the time point 72 h. The false discovery rate (FDR)-adjusted *p* value was used to estimate representative DEGs using the Benjamini-Hochberg method. Genes with *p* < 0.05 and fold change > 2 were considered to be differentially expressed. All DEGs in the four treatment groups were taken together, and their corresponding normalized signal values in P78, P75, T25, T31 and the control were further subjected to hierarchical clustering analysis using Cluster 3.0 and Java TreeView software by the average linkage of hierarchical cluster analysis. The fold changes of unigenes in P78, P75, T25 and T31 were obtained by comparing the normalized signal values with that in the control. To identify the underlying pathways responding to elevated CO_2_ and temperature, Kyoto Encyclopedia of Genes and Genomes (KEGG) pathway enrichment analyses of the DEGs in the four treatments (P78, P75, T25 and T31) were performed in the pathway database (http://www.genome.jp/kegg/). Fisher’s exact tests were implemented to determine DEG enrichment. The FDR-adjusted *p* value was used to estimate the most representative pathways using the Benjiamini-Hochberg method with *p* < 0.05.

In addition to KEGG pathways, specific categories were classified artificially according to references^30^,^60^ in which have gene enrichment analyses for transcriptome or microarray results were successfully performed to reveal the responses of marine organisms to changes in the seawater environment. The genes involved in these reported functional categories were searched for in our microarray results for the four treatments (P78, P75, T25 and T31), and the fold changes in the expression of the matched genes are listed individually. Genes with *p* < 0.05 and a fold change > 2 were considered to be significantly differentially expressed. The category enriched with these genes was considered to be the principal category. These categories of specific genes serve as a supplement for the KEGG pathway analysis.

### Statistical analyses

All the physiological, biochemical and morphological data analyses were performed using SPSS version 18.0 for Windows (SPSS Inc., Chicago, IL, USA). The results of seawater chemistry, ALP activity, NCR, calcium content and amino acid content were analyzed by One-way ANOVA to test for significant differences between each treatment and the control. The figures were drawn using SigmaPlot version 12.5 (Systat Software, San Jose, CA, USA) and Origin version 7.5 (Originlab, Northampton, MA, USA). All of the results are presented as the means and standard deviation.

### Data Availability

The probe sequences and other details on the microarray in this study have been deposited in the GEO database and Dryad database, and are accessible through the GEO Series Accession Number GSE57171 or Dryad: doi:10.5061/dryad.958sr.

## Additional Information

**How to cite this article**: Li, S. *et al*. Transcriptome and Biomineralization Responses of the Pearl Oyster *Pinctada fucata* to Elevated CO_2_ and Temperature. *Sci. Rep*. **6**, 18943; doi: 10.1038/srep18943 (2016).

## Supplementary Material

Supplementary Information
