# Supplementary material for: Transcriptome and biomineralization responses of the pearl oyster Pinctada fucata to elevated CO2 and temperature
Source: Sci Rep. 2016 Jan 6;6:18943. doi: 10.1038/srep18943 (PMC4702168; doi:10.1038/srep18943)
Supplement: Supplementary Information [file srep18943-s1.pdf]

Transcriptome and biomineralization responses of the pearl oyster *Pinctada fucata* to elevated CO_2 and temperature

Shiguo Li[1], Chuang Liu[1,2], Jingliang Huang[1], Yangjia Liu[1], Shuwen Zhang[1], Guilan Zheng[1], Liping Xie[1*] & Rongqing Zhang[1*]

[1]Institute of Marine Biotechnology, Collaborative Innovation Center of Deep Sea Biology, School of Life Sciences, Tsinghua University, Beijing 100084, China, [2]Tsinghua-Peking Joint Center for Life Sciences, School of Life Sciences, Tsinghua University, Beijing 100084, China.

*Corresponding authors:

Dr. Rongqing Zhang

rqzhang@tsinghua.edu.cn

Dr. Liping Xie

lpxie@tsinghua.edu.cn.

Methods

Liquid chromatography-tandem mass spectrometry (LC-MS/MS)

A standard solution was prepared by dissolving the amino acid standards in acetonitrile. All the standards and other reagents were obtained from Sigma-Aldrich (St. Louis, MO, USA, ≥99.99% purity). The stock standard mixture (10 μg mL^{-1}) was prepared by diluting the standard solutions, from which the calibration standards with a range of 5–2500 ng.mL^{-1} were prepared by serially diluting the mixture with acetonitrile.

The mantles from 10 pearl oysters in each tank were collected and pooled together to obtain one biological replicate. A homogeneous 1.0 g sample was hydrolyzed by 6 M hydrochloric acid (HCl) at 110°C for 14 h. The hydrolysate was cooled to room temperature and HCl was added to obtain a total volume of 10 mL. One mL of the hydrolysate was lyophilized, redissolved by 2.0 mL of 0.1 M HCl, and stored at 4°C until analysis. Before determination, the sample was mixed for 3 min and centrifuged at 10,000 × *g* for 5 min at room temperature. The amino acid content was determined with a 1290 analytical HPLC system connected to a 6460 triple quadrupole mass spectrometer (LC-MS/MS, Agilent Technologies, Santa Clara, CA, USA). A 10-μL sample was injected through an auto sampler. The separation was collected with a C_{18} column (Perkin-Elmer, 4.6 mm × 250 mm, 5 μm). The mobile phase was composed of (A) acetonitrile and (B) acetic acid sodium acetate buffer (0.03 M sodium acetate, 0.15% triethylamine, pH 5.2). The column temperature was 40°C and the determination wavelength was 360 nm. The equation obtained from the standard solutions was used for the sample analysis. The results were presented as amino acids mg/g mantle ± SD.

Microarray experiment

Total RNA of the mantle samples that were collected from pearl oysters at 72 h in the two CO_2 treatments (P78 and P75), the two temperature treatments (T25 and T31) and the control were extracted using TRIZOL Reagent (Life technologies, Carlsbad, CA, US) following the manufacturer's instructions and further purified using the NucleoSpin RNA clean-up kit (Macherey-Nagel, Duren, Germany). The RNA samples were quantified by measuring absorbance ratios using a ND-1000 spectrophotometer (NanoDrop Technologies, Wilmington, DE, USA) and electrophoresed on a 1.0% formaldehyde-denatured agarose gel to determine their integrity. RNA labelling and microarray hybridization were conducted by the CapitalBio Corp (Beijing, China) following

the previously reported protocol [1]. Dye-labeled cDNA was created using Cy3 dye (GE Healthcare Cat. No. PA53021), and then hybridized with the microarray at 45 °C for 15 h. After hybridization, the slides were rinsed with washing solution (0.2% SDS, 2 × SSC) for 5 min at 42 °C and washed again with washing solution (2 × SSC) for 5 min at room temperature. The dried slides were scanned using an Agilent G2565CA microarray scanner system (Agilent technologies, Berkshire, UK). Agilent's Feature Extraction Software was used for the microarray image analyses in order to convert the image signal into the digital signal. Signal intensity of each microarray was normalized using GeneSpring GX version 11.0.2 software (Agilent Technologies) with the percentile shift method.

To validate the microarray results, a set of 14 unigenes were selected randomly in the microarray for real-time quantitative PCR (RT-qPCR) analyses. Total RNA of the mantle samples was extracted using the protocol as described above. The RT-qPCR was performed with 1 μL of cDNA, 0.4 μM of each primer and 2 × SYBR Green Master Mix using SYBR® *Premix Ex Taq*™ II (Takara, Japan) to obtain a total volume of 20 μL. All PCR reactions were run as follows: 1 cycle of 95 °C for 30 s, 40 cycles of 95 °C for 5 s, 60 °C for 30 s, 1 cycle of 95 °C for 15 s, 60 °C for 1 min and 95 °C for 15 s. The fluorescent products were detected using StepOnePlus™ Real-Time PCR system (Applied Biosystems, Foster, CA, US). The reference housekeeping gene ACTIN [2] was used as the internal control and the $2^{-\Delta\Delta CT}$ method [3] was used to analyze the relative fold change in gene expression. The correlation coefficients (R^2) between the results of gene expression levels of microarray and RT-qPCR were calculated. Information regarding these unigenes, primers and accession numbers in RT-qPCR is listed in Supplementary Table 1.

Supplementary tables

Supplementary Table 1. Amino acid contents in the mantle of *Pinctada fucata* exposed to CO_2 and temperature stress (Mean ± SD). *: significant difference ($p < 0.05$). **: significant difference ($p < 0.01$).

**Amino acid**	**Amino acid content in treatment ($mg \cdot g^{-1}$)**				
	Control	P78	P75	T25	T31
**Phe**	8.45 ± 0.37	25.04 ± 3.12 **	37.18 ± 0.92**	9.07 ± 0.89	8.91 ± 0.70
**Cys**	1.80 ± 0.47	2.21 ± 1.17*	4.06 ± 0.33**	2.12 ± 0.98	1.76 ± 0.79
**Tyr**	56.64 ± 0.14	47.94 ± 5.23**	11.37 ± 3.95**	57.96 ± 0.66	53.38 ± 0.46
**Met**	6.67 ± 0.26	7.40 ± 0.96*	30.58 ± 2.56**	6.79 ± 0.78	6.01 ± 0.58
**Ala**	24.01 ± 1.00	30.48 ± 7.31**	98.87 ± 7.33**	24.37 ± 1.52	23.45 ± 1.33
**Glu**	232.09 ± 19.58	216.91 ± 20.46*	74.25 ± 2.78**	240.38 ± 20.18	230.14 ± 34.20
**Gly**	195.04 ± 6.14	160.11 ± 2.64**	54.52 ± 10.21**	196.36 ± 6.67	193.04 ± 6.47
**Arg**	21.43 ± 1.23	22.01 ± 1.93	22.77 ± 2.52	22.76 ± 1.74	19.80 ± 1.55
**Asn**	4.08 ± 0.83	4.20 ± 0.25	4.03 ± 0.58	4.40 ± 0.22	3.97 ± 0.34
**Gln**	0.63 ± 0.04	0.59 ± 0.07	0.69 ± 0.12	0.70 ± 0.16	0.58 ± 0.08

Supplementary Table 2. List of candidate genes and associated primers used for the RT-qPCR. The fold changes of the selected unigenes from the microarray are listed in the table. The values in parentheses represent the fold changes of the unigenes confirmed by RT-qPCR. Positive and negative numbers represent up-regulation and down-regulation of the unigenes, respectively.

Probe name	Gene symbol	Description	Accession number	Primer sequence	Gene expression pattern (fold change)			
					P78	P75	T25	T31
**CUST_53622**	P14	Prismalin-14	AB159512.1	TGGGTATGGCGGATTTAACGGTG<br>AATCCGCCATCATCGTCACCAAA	-1.33<br>(-1.00)	-2.10<br>(-3.33)	-1.66<br>(-2.32)	-10.22<br>(-4.35)
**CUST_49427**	P39	Prisilkin-39	EU921665.1	TAGCTAGTGGTGGTTTAGGTGCC<br>TACCCGCTATATCCGCCGTAACC	-1.65<br>(-1.11)	-1.08<br>(-0.56)	-1.84<br>(-2.94)	-2.20<br>(-1.23)
**CUST_47372**	SHE7	Shematrin-7	AB244425	TGGAGCCGGAAGTTTGTCATCT<br>CCGAGGTTTACTGATGGTCCGT	13.17<br>(6.48)	17.67<br>(8.25)	3.54<br>(4.25)	-2.90<br>(-1.33)
**CUST_52107**	SHE6	Shematrin-6	AB244424	ACACCTTCGGAACATTGGCAAC<br>CACCTCCAAGACCGAGACTGAG	-1.34<br>(-2.02)	-3.72<br>(-2.91)	-6.41<br>(-6.34)	-4.80<br>(-5.55)
**CUST_54973**	KRMP5	Lysine-rich matrix protein 5	ABO87298.1	TCACCCTTGGGATTGGAAATGCA<br>GCCAAAGTTGTAATCATCGCCACC	-2.10<br>(- 3.33)	-1.18<br>(-1.29)	1.02<br>(0.69)	-1.44<br>(-1.89)
**CUST_54904**	NAC	Nacrein	D83523.1	GAGCCAGAGGATGGGGAAA<br>GCCTCCATAGGTGAAACGA	-15.17<br>(-11.06)	-1.98<br>(-2.67)	-1.71<br>(-1.32)	-6.27<br>(-4.75)
**CUST_57088**	PIF80	Pif80	AB236929.1	TGCTGCCATCACGTGAGTATG<br>GACTTCCCTTTCTCACACTTCCA	-4.39<br>(-3.04)	-5.44<br>(-3.57)	-3.81<br>(-4.51)	-2.84<br>(-2.92)
**CUST_45506**	MG1	Mantle gene 1	DQ104255	GTCCTTGGCGTCGTAGTTTGTG<br>TCCTCCTCTCCCTATTTCCGGT	10.14<br>(7.59)	7.57<br>(5.18)	7.12<br>(7.53)	6.06<br>(3.04)
**CUST_57043**	LUSA	Lustrin A	AAB95154.1	AGCCACTAGTAGCAGCTTCTCG<br>TAGGGGAAGGTTGCTGTTGAGG	1.83<br>(2.34)	1.62<br>(0.51)	1.29<br>(1.06)	-1.24<br>(-0.28)
**CUST_27219**	PEA	Pearlin	AB020779	CCTGGAATCATGACGTGCACAC<br>AGCAGTATGAGTAACGTCCGCA	1.06<br>(2.33)	-3.42<br>(5.07)	1.76<br>(0.95)	-2.42<br>(-3.59)
**CUST_33106**	ACCBP1	Amorphous calcium carbonate binding protein 1	DQ473430.1	ACTGCTGTCCCGAACCGTATTA<br>TCAGGTTCCTCAGACATTGCCT	3.21<br>(2.20)	1.19<br>(1.39)	1.28<br>(1.88)	-1.71<br>(-1.82)
**CUST_56098**	CALP	Calponin	AB735601	CAGGCAGGCATGAGAGGTTTTG<br>TTTGCTGCATTCTGAGCTGCTC	1.75<br>(1.65)	1.41<br>(1.90)	1.14<br>(0.48)	-1.59<br>(-1.01)
**CUST_50654**	CAM	Calmodulin	AY341376	ATCCAACCGAGGCTGAACTTCA<br>GTGTCTTTCATCTTTCGCGCCA	2.49<br>(1.44)	-2.64<br>(-3.63)	-4.45<br>(-3.85)	-3.67<br>(-2.13)
**CUST_57417**	CALR	Calreticulin	EF551334.1	ACAAGCCAGAACACATCCCTGA<br>CCATTCTCCCTTGTACTCGGGG	3.48<br>(2.03)	1.09<br>(1.20)	1.16<br>(1.58)	1.23<br>(0.88)
---	ACT	Actin	AB252571.1	TACCGCCGCGTCATCATCAT<br>TGCCTCGGGACATCTGAACC	---	---	---	---

Supplementary Table 3. KEGG pathway analysis for unigenes with significant differential expression (p < 0.05 and fold change > 2) in *Pinctada fucata* exposed to elevated CO_2 and temperature. DEGs: Differentially expressed genes.

Treatment	Category	Pathway	Genes in Term	DEGs	Up-regulated DEGs	Down-regulated DEGs	*p*-value
**P78**	Amino acid metabolism	ko00360://Phenylalanine metabolism	145	29	12	17	0.039395365
**P75**	Amino acid metabolism	ko00360://Phenylalanine metabolism	145	32	7	25	0.020621893
		ko00270://Cysteine and methionine metabolism	81	22	10	12	0.020621893
		ko00400://Tyrosine and tryptophan biosynthesis	17	8	1	7	0.020621893
		ko00410://beta-Alanine metabolism	92	22	5	17	0.029665796
	Immune system	ko04620://Toll-like receptor signaling pathway	149	31	14	17	0.030709777
**T25**	Lipid metabolism	ko00590://Arachidonic acid metabolism	151	23	6	17	0.003058989
	Immune system	ko04620://Toll-like receptor signaling pathway	149	24	17	7	0.030709777
	Glycan biosynthesis and metabolism	ko00601://Glycosphingolipid biosynthesis	42	9	1	8	0.04576765
**T31**	Transport and catabolism	ko04142://Lysosome	394	52	44	8	0.00363459
	Glycan biosynthesis and metabolism	ko00511://Other glycan degradation	62	15	12	3	0.00363459

Supplementary Table 4. Fold changes in gene expressions of specific interest in *Pinctada fucata* exposed to elevated CO_2 and temperature. The asterisk indicates the genes that expressed no significant change (fold change < 2 or $p > 0.05$).

Category	Probe Name	Unigene	Gene symbol	Function-Description	Gene expression (fold change > 2 and $p < 0.05$)			
					**P78**	**P75**	**T25**	**T31**
**Ion and acid-base regulation**	CUST_18035_PI429347424	Unigene18045_TP	V-ATPase	V-type proton ATPase subunit E 1	7.27	6.25	*	*
	CUST_18034_PI429347424	Unigene18044_TP	V-ATPase	V-type proton ATPase subunit E 1	2.86	6.25	*	*
	CUST_39438_PI429347424	Unigene39466_TP	V-ATPase	V-type proton ATPase catalytic subunit A	2.87	3.21	*	*
	CUST_45016_PI429347424	Unigene45050_TP	V-ATPase	V-type proton ATPase 16 kDa proteolipid subunit	*	2.60	*	*
	CUST_48645_PI429347424	Unigene48682_TP	V-ATPase	V-type proton ATPase subunit c	2.46	2.04	*	*
	CUST_50619_PI429347424	Unigene50658_TP	V-ATPase	V-type proton ATPase catalytic subunit A	3.15	2.65	*	*
	CUST_54425_PI429347424	Unigene54465_TP	V-ATPase	V-type proton ATPase subunit C 1-A	2.18	3.54	*	*
	CUST_55456_PI429347424	Unigene55496_TP	V-ATPase	V-type proton ATPase catalytic subunit A	3.46	7.21	*	*
	CUST_55635_PI429347424	Unigene55675_TP	V-ATPase	V-type proton ATPase 21 kDa proteolipid subunit	2.49	3.01	*	*
	CUST_56391_PI429347424	Unigene56431_TP	V-ATPase	V-type proton ATPase 16 k Da proteolipid subunit	2.18	2.42	*	*
	CUST_57255_PI429347424	Unigene57295_TP	V-ATPase	V-type proton ATPase subunit C 1-A	*	2.20	*	*
	CUST_43429_PI429347424	Unigene43460_TP	V-ATPase	V-type proton ATPase subunit F 1	2.93	*	*	*
	CUST_54640_PI429347424	Unigene54680_TP	V-ATPase	V-type proton ATPase subunit E	2.59	2.06	*	*
	CUST_55387_PI429347424	Unigene55427_TP	V-ATPase	V-type proton ATPase catalytic subunit A	3.01	*	*	2.96
	CUST_45926_PI429347424	Unigene45960_TP	NKA	Sodium/potassium-transporting ATPase subunit alpha	2.51	*	*	2.48
	CUST_21437_PI429347424	Unigene21452_TP	NKA	Sodium/potassium-transporting ATPase subunit alpha	*	*	*	2.58
	CUST_15255_PI429347424	Unigene15263_TP	NHE5	Sodium/hydrogen exchanger 5	2.50	2.82	3.17	*
	CUST_28238_PI429347424	Unigene28257_TP	NHE3	Sodium/hydrogen exchanger 3	4.76	4.89	7.80	*
	CUST_31582_PI429347424	Unigene31603_TP	CLC	Similar to CLC chloride channel	4.70	*	*	*
	CUST_26096_PI429347424	Unigene26113_TP	NCKX1	Similar to sodium/potassium/calcium exchanger 1	2.30	2.81	*	*
	CUST_4924_PI429347424	Unigene4926_TP	NCKX1	Sodium/potassium/calcium exchanger 1	*	4.01	*	*
	CUST_35_PI429347424	Unigene35_TP	NCKX6	Sodium/potassium/calcium exchanger 6	2.90	*	*	*
	CUST_54840_PI429347424	Unigene54880_TP	NBC1	Sodium bicarbonate cotransporter 1	*	*	*	*
	CUST_55044_PI429347424	Unigene55084_TP	NBC3	Sodium bicarbonate cotransporter 3	*	*	*	*
	CUST_51999_PI429347424	Unigene52038_TP	NBC1	Sodium bicarbonate cotransporter 1	*	*	*	*

	CUST_52787_PI429347424	Unigene52826_TP	NBC3	Sodium bicarbonate cotransporter 3	*	*	*	*
	CUST_35106_PI429347424	Unigene35128_TP	NBC3	Sodium bicarbonate cotransporter 3	*	*	*	*
	CUST_12081_PI429347424	Unigene12089_TP	SLC26A3	Anion exchanger,solute carrier family 26 member 3	-6.87	*	*	*
	CUST_57515_PI429347424	Unigene57555_TP	SLC26A6	Anion exchanger,solute carrier family 26 member 6	-4.74	*	*	*
	CUST_51946_PI429347424	Unigene51985_TP	SLC26A4	Pendrin, Solute carrier family 26 member 4	-3.63	*	*	*
	CUST_26039_PI429347424	Unigene26056_TP	VGCC	Voltage-dependent calcium channel subunit alpha-2/delta-4	-2.77	-2.60	*	*
	CUST_58410_PI429347424	Unigene58450_TP	SERCA	Sarco/endoplasmic reticulum calcium ATPase isoform C	*	2.26	*	*
	CUST_33162_PI429347424	Unigene33183_TP	AE2	AE-like protein	*	*	*	*
	CUST_34899_PI429347424	Unigene34921_TP	AE2	AE-like protein	*	*	*	*
**Heat shock response and protein homeostasis**	CUST_27509_PI429347424	Unigene27527_TP	HSP70	Heat shock 70 kDa protein 12B	2.99	2.89	*	*
	CUST_57889_PI429347424	Unigene57929_TP	HSP70	Heat shock 70 kDa protein 4	*	3.56	*	*
	CUST_12501_PI429347424	Unigene12509_TP	HSP70	Heat shock 70 kDa protein 12A	*	8.63	*	*
	CUST_48599_PI429347424	Unigene48636_TP	HSP70	Heat shock 70 kDa protein 12A	2.55	*	2.95	*
	CUST_50857_PI429347424	Unigene50896_TP	HSP70	Heat shock 70 kDa protein 12A	2.61	*	2.15	*
	CUST_4488_PI429347424	Unigene4490_TP	HSP70	similar to heat shock 70kDa protein 12A	*	*	*	3.43
	CUST_13483_PI429347424	Unigene13491_TP	HSP70	similar to Heat shock 70 kDa protein 12B	*	*	*	2.84
	CUST_57881_PI429347424	Unigene57921_TP	HSP90	Heat shock protein HSP 90-beta	*	2.14	*	*
**Oxidation**	CUST_21268_PI429347424	Unigene21282_TP	CYP450	Cytochrome P450 4B1	*	*	3.48	*
	CUST_53747_PI429347424	Unigene53786_TP	CYP450	Cytochrome P450 2J6	*	*	11.96	*
	CUST_33912_PI429347424	Unigene33934_TP	CYP450	Cytochrome P450 2C28	*	*	4.5	*
	CUST_3084_PI429347424	Unigene3085_TP	CYP450	Cytochrome P450 2D14	*	*	12.23	*
	CUST_839_PI429347424	Unigene839_TP	CYP450	Cytochrome P450 2C20	*	3.35	7.97	*
	CUST_30347_PI429347424	Unigene30368_TP	CYP450	cytochrome P450 3A4	*	*	3.87	7.61
	CUST_45674_PI429347424	Unigene45708_TP	CYP450	cytochrome P450, family 3, subfamily c	*	*	*	6.27
	CUST_13911_PI429347424	Unigene13919_TP	CYP450	cytochrome P450 3A2	*	*	*	7.14
	CUST_54667_PI429347424	Unigene54707_TP	CYP450	cytochrome P450	*	*	*	6.00
**Anti-oxidation**	CUST_27372_PI429347424	Unigene27390_TP	GST1	Glutathione S-transferase 1	*	-6.86	*	*
	CUST_48632_PI429347424	Unigene48669_TP	GSTM5	Glutathione S-transferase Mu 5	*	-6.42	*	*
	CUST_25915_PI429347424	Unigene25932_TP	GST	Glutathione S-transferase sigma class protein	*	*	2.44	*
	CUST_55989_PI429347424	Unigene56029_TP	GPO	Non-selenium glutathione peroxidase	*	*	2.47	*

**Apoptosis**	CUST_7652_PI429347424	Unigene7657_TP	CAS8	Caspase-8	*	*	*	3.02
	CUST_39366_PI429347424	Unigene39394_TP	CAS3C	Similar to caspase 3C	*	*	-2.95	*
	CUST_51165_PI429347424	Unigene51204_TP	CAS3C	Caspase 3C	*	*	*	*
	CUST_5294_PI429347424	Unigene5296_TP	CAS7	Caspase-7	*	*	*	*
	CUST_27929_PI429347424	Unigene27947_TP	CAS7	Caspase-7	*	*	-4.84	*
	CUST_54168_PI429347424	Unigene54208_TP	CAS2	Caspase-2	*	*	*	*
	CUST_3250_PI429347424	Unigene3251_TP	CAS2	Caspase-2	*	*	*	*
	CUST_57368_PI429347424	Unigene57408_TP	CAS10	Caspase-10	*	*	*	*
	CUST_48089_PI429347424	Unigene48126_TP	CAS6	Caspase-6	*	*	*	*
	CUST_7798_PI429347424	Unigene7804_TP	TNFR16	Tumor necrosis factor receptor superfamily member 16	-3.56	-3.27	*	*
**Cell division**	CUST_34707_PI429347424	Unigene34729_TP	QN1	Chromosome segregation ATPases QN1 homolog	-2.73	-2.72	*	*
	CUST_15610_PI429347424	Unigene15618_TP	RAD50	DNA repair protein RAD50	*	-2.63	*	*
	CUST_1771_PI429347424	Unigene1772_TP	MSH2	DNA mismatch repair protein Msh2	*	-3.23	*	*
	CUST_58413_PI429347424	Unigene58453_TP	PCLO	Protein piccolo	*	-2.08	*	*
	CUST_50889_PI429347424	Unigene50928_TP	RFC	Replication factor C subunit 2	*	-4.25	*	*
	CUST_14834_PI429347424	Unigene14842_TP	MCM6	Zygotic DNA replication licensing factor mcm6	*	-4.09	*	*
	CUST_58121_PI429347424	Unigene58161_TP	SPTB	Spectrin beta chain	*	-2.01	*	*
	CUST_26910_PI429347424	Unigene26928_TP	NESP1	Nesprin-1	*	-2.23	*	*
	CUST_11750_PI429347424	Unigene11758_TP	GAS8	Growth arrest-specific protein 8	*	-3.22	*	*
	CUST_26641_PI429347424	Unigene26659_TP	CCDC146	Coiled-coil domain-containing protein 146	*	-2.13	*	*
	CUST_432_PI429347424	Unigene432_TP	INT	Interaptin	*	-4.10	*	*
	CUST_54660_PI429347424	Unigene54700_TP	LRR	Putative leucine-rich repeat-containing protein	*	-4.12	*	*
	CUST_33748_PI429347424	Unigene33769_TP	AK7	Putative adenylate kinase 7	*	-2.25	*	*
	CUST_16450_PI429347424	Unigene16458_TP	ATAD2B	ATPase family AAA domain-containing protein 2B	-2.08	-2.14	*	*
	CUST_34364_PI429347424	Unigene34386_TP	CCDC151	Coiled-coil domain-containing protein 151	*	-4.51	*	*
	CUST_23601_PI429347424	Unigene23616_TP	ABCC3	Canalicular multispecific organic anion transporter 2	-2.17	-2.03	*	*
	CUST_23242_PI429347424	Unigene23257_TP	CCDC39	Coiled-coil domain-containing protein 39	*	-2.83	*	*
	CUST_22950_PI429347424	Unigene22965_TP	NP58	Nucleolar protein 58	*	-3.57	*	*
	CUST_8593_PI429347424	Unigene8599_TP	AKAP9	A-kinase anchor protein 9	*	-4.58	*	*
	CUST_211_PI429347424	Unigene211_TP	MSH2	DNA mismatch repair protein Msh2	*	-3.62	*	*

	CUST_9741_PI429347424	Unigene9748_TP	ODF2	Outer dense fiber protein 2	*	-2.24	*	*
	CUST_46591_PI429347424	Unigene46625_TP	RAD50	DNA repair protein RAD50	-2.19	-2.08	*	*
	CUST_4158_PI429347424	Unigene4160_TP	ANKRD26	Ankyrin repeat domain-containing protein 26	-2.52	-2.16	*	*
	CUST_8502_PI429347424	Unigene8508_TP	AKAP9	A-kinase anchor protein 9	*	-2.20	*	*
	CUST_34034_PI429347424	Unigene34056_TP	CNTRL	Centriolin	-2.24	-2.35	*	*

Supplementary Table 5. The expressions of energy production related genes in *Pinctada fucata* exposed to elevated CO_2 and temperature. The asterisk indicates the genes that expressed no significant change (fold change < 2 or $p > 0.05$).

Catergory	ProbeName	Unigene	Gene symbol	Function-Description	Gene expression (fold change > 2 and $p < 0.05$)			
					P78	P75	T25	T31
Oxidative phosphorylation	CUST_56391_PI429347424	Unigene56431_TP	F-ATPase	F0F1-type ATP synthase, subunit c	2.18	2.42	*	*
	CUST_48645_PI429347424	Unigene48682_TP	F-ATPase	F0F1-type ATP synthase, subunit c	2.46	2.04	*	*
	CUST_55635_PI429347424	Unigene55675_TP	F-ATPase	F0F1-type ATP synthase, subunit c	2.49	3.01	*	*
	CUST_53845_PI429347424	Unigene53884_TP	CI	NADH dehydrogenase [ubiquinone] flavoprotein 1	*	-2.95	*	*
	CUST_54324_PI429347424	Unigene54364_TP	CI	Probable NADH dehydrogenase [ubiquinone] 1 alpha	*	-2.38	*	*
	CUST_52302_PI429347424	Unigene52341_TP	CI	NADH-ubiquinone oxidoreductase 75 kDa subunit	2.50	*	*	*
	CUST_58013_PI429347424	Unigene58053_TP	CII	Succinate dehydrogenase [ubiquinone] iron-sulfur subunit	*	*	*	*
	CUST_32570_PI429347424	Unigene32591_TP	CII	Succinate dehydrogenase cytochrome b560 subunit	*	*	*	*
	CUST_58280_PI429347424	Unigene58320_TP	CII	Succinate dehydrogenase [ubiquinone] flavoprotein subunit B	*	*	*	*
	CUST_32734_PI429347424	Unigene32755_TP	CIII	Ubiquinol-cytochrome c reductase complex chaperone CBP3	*	*	*	*
	CUST_57937_PI429347424	Unigene57977_TP	CIII	Cytochrome b-c1 complex subunit Rieske, mitochondrial	*	*	*	*
	CUST_54083_PI429347424	Unigene54122_TP	CIV	Cytochrome c oxidase subunit 4 isoform 2	*	*	*	*
	CUST_30475_PI429347424	Unigene30496_TP	CIV	similar to cytochrome oxidase assembly factor	*	*	*	*
	CUST_56791_PI429347424	Unigene56831_TP	CIV	cytochrome c oxidase polyprotein Vb	*	*	*	*
	CUST_44904_PI429347424	Unigene44938_TP	AC	Adenylate cyclase	2.58	*	*	*
	CUST_11250_PI429347424	Unigene11258_TP	PKA	Subunit of protein kinase A	2.42	*	*	*
Glycolysis	CUST_56281_PI429347424	Unigene56321_TP	K6PF	6-phosphofructokinase	-3.98	-7.93	-4.25	*
	CUST_36097_PI429347424	Unigene36119_TP	PKM	Pyruvate kinase	*	-2.08	*	*
TCA	CUST_52814_PI429347424	Unigene52853_TP	ACLY	ATP-citrate synthase	*	*	*	*
	CUST_25626_PI429347424	Unigene25643_TP	IDH	Isocitrate dehydrogenase [NAD] subunit gamma	*	*	*	*
	CUST_8921_PI429347424	Unigene8927_TP	ODH	Probable 2-oxoglutarate dehydrogenase E1 component DHKTD1 homolog	5.05	-2.40	-4.84	*
	CUST_18014_PI429347424	Unigene18024_TP	ODH	Probable 2-oxoglutarate dehydrogenase E1 component DHKTD1	*	-2.69	-3.59	*
Fatty acid and glycerol	CUST_35118_PI429347424	Unigene35140_TP	ACLS1	Acyl-CoA synthetase long-chain family member 1	5.16	4.36	*	*

**metabolism**	CUST_27687_PI429347424	Unigene27705_TP	ACO3	Acyl-coenzyme A oxidase 3/Acyl-CoA dehydrogenases	2.43	*	*	*
	CUST_22524_PI429347424	Unigene22539_TP	ACOX	Palmitoyl-CoA oxidase	2.33	*	*	*
	CUST_222_PI429347424	Unigene222_TP	ACOX3	Pristanoyl acyl-Coenzyme A oxidase 3	2.53	*	*	*
	CUST_24288_PI429347424	Unigene24304_TP	LCAD	Long-chain acyl-CoA dehydrogenase	3.24	*	*	*
	CUST_37384_PI429347424	Unigene37408_TP	GK	Glycerol kinase	-2.42	*	*	*

Supplementary Table 6. The expression changes of biomineralization-related genes form the microarray. The positive and negative numbers represent up-regulation and down-regulation of the unigenes, respectively. The asterisk indicates the genes that expressed no significant change (fold change < 2 or *p* > 0.05). The rows with grey shading represent the unigenes with no significant change (fold change < 2 or *p* > 0.05) in all the four treatments.

Unigene	Probe name	Description	Gene symbol	Gene expression (fold change > 2 and *p* < 0.05)			
				P78	P75	T25	T31
**Unigene50937_TP**	CUST_50898_PI429347424	Shell matrix protein	SMP	*	2.24	-2.53	*
**Unigene27237_TP**	CUST_27219_PI429347424	Matrix protein N16.5	N16	*	-3.14	*	-2.42
**Unigene38603_TP**	CUST_38575_PI429347424	Lysine-rich matrix protein 1	LRMP	3.04	2.68	-4.48	-6.35
**Unigene44100_TP**	CUST_44067_PI429347424	Shell matrix protein	SMP	*	*	*	*
**Unigene1709_TP**	CUST_1708_PI429347424	Mantle protein 11	MP11	2.88	*	*	5.24
**Unigene38692_TP**	CUST_38664_PI429347424	Mantle protein 9	MP9	-2.66	-3.69	*	-4.44
**Unigene56023_TP**	CUST_55983_PI429347424	Mantle gene 4	MG4	*	*	*	*
**Unigene45540_TP**	CUST_45506_PI429347424	Mantle gene 1	MG1	10.14	7.57	7.12	6.06
**Unigene56810_TP**	CUST_56770_PI429347424	Veliger mantle 1	VM1	-3.21	-6.70	-3.84	-2.87
**Unigene54789_TP**	CUST_54749_PI429347424	Mantle gene 4	MG4	4.40	2.84	*	*
**Unigene53901_TP**	CUST_53862_PI429347424	Mantle gene 3	MG3	-3.35	*	*	*
**Unigene44462_TP**	CUST_44428_PI429347424	Mantle gene 8	MG8	*	*	*	*
**Unigene53661_TP**	CUST_53622_PI429347424	Prismalin-14 precursor	P14	*	-2.10	*	-10.22
**Unigene49464_TP**	CUST_49427_PI429347424	Prisilkin-39	P39	*	*	*	-2.20
**Unigene47407_TP**	CUST_47372_PI429347424	Shematrin-7	SHE7	13.17	17.67	3.54	-2.90
**Unigene55013_TP**	CUST_54973_PI429347424	Lysine-rich matrix protein 5	KRMP-5	-2.10	*	*	*
**Unigene47710_TP**	CUST_47674_PI429347424	Lysine-rich matrix protein 7	KRMP-7	*	*	*	*
**Unigene54899_TP**	CUST_54859_PI429347424	Pif	PIF	*	*	*	-3.04
**Unigene57128_TP**	CUST_57088_PI429347424	Pif	PIF	-4.39	-5.44	-3.81	-2.84
**Unigene62_TP**	CUST_62_PI429347424	Pif	PIF	*	*	-4.00	-3.55
**Unigene57083_TP**	CUST_57043_PI429347424	Lustrin A	LUSA	*	*	*	*
**Unigene27237_TP**	CUST_27219_PI429347424	Pearlin	PEA	*	-3.42	*	-2.42
**Unigene33127_TP**	CUST_33106_PI429347424	Amorphous calcium carbonate binding protein 1	ACCBP1	*	*	*	*

**Unigene28008_TP**	CUST_27989_PI429347424	Calponin	CALP	*	-2.98	*	*
**Unigene56138_TP**	CUST_56098_PI429347424	Calponin-like protein	CALPL	*	*	*	*
**Unigene51236_TP**	CUST_51197_PI429347424	Calponin-like protein	CALPL	*	*	*	*
**Unigene50693_TP**	CUST_50654_PI429347424	EF hand family protein	EF	*	-2.20	*	*
**Unigene57457_TP**	CUST_57417_PI429347424	Calreticulin	CALR	3.48	*	*	*
**Unigene38419_TP**	CUST_38391_PI429347424	Tyrosinase	TYR	*	-7.04	-4.11	-9.97
**Unigene57981_TP**	CUST_57941_PI429347424	Tyrosinase	TYR	*	-7.54	-3.69	-8.29
**Unigene47447_TP**	CUST_47412_PI429347424	Tyrosinase	TYR	*	*	*	*
**Unigene13528_TP**	CUST_13520_PI429347424	Tyrosinase	TYR	*	-29.87	-4.82	-5.09
**Unigene31962_TP**	CUST_31941_PI429347424	Tyrosinase-like protein 1	TYRL1	2.43	2.81	*	*
**Unigene11160_TP**	CUST_11152_PI429347424	Tyrosinase-like protein 1	TYRL1	2.77	3.62	*	*
**Unigene58124_TP**	CUST_58084_PI429347424	Tyrosinase-like protein 2	TYRL2	4.51	3.54	*	*
**Unigene7580_TP**	CUST_7575_PI429347424	Tyrosinase-like protein 2	TYRL2	*	*	*	2.10
**Unigene27084_TP**	CUST_27066_PI429347424	Shell protein 10	SP10	*	8.12	2.55	*
**Unigene47469_TP**	CUST_47434_PI429347424	Tyrosinase-like protein	TYRL	*	*	*	*
**Unigene54944_TP**	CUST_54904_PI429347424	Nacrein	NAC	-15.17	*	-6.27	*
**Unigene24820_TP**	CUST_24804_PI429347424	Shematrin-4	SHE4	13.29	4.51	-4.58	*
**Unigene31_TP**	CUST_31_PI429347424	Shematrin-5	SHE5	*	-5.74	-11.28	-30.88
**Unigene52146_TP**	CUST_52107_PI429347424	Shematrin-6	SHE6	*	-3.72	-6.41	-4.80
**Unigene32248_TP**	CUST_32227_PI429347424	Alkaline phosphatase	ALP	-2.25	-3.27	*	-2.18
**Unigene58009_TP**	CUST_57969_PI429347424	Carbonic anhydrase precursor	CA	-11.57	-27.34	*	*
**Unigene56730_TP**	CUST_56690_PI429347424	Galectin	GAL	2.18	2.89	*	*
**Unigene57196_TP**	CUST_57165_PI429347424	Astacin-like prtein	ASLP	-2.01	-4.11	*	-2.54
**Unigene58007_TP**	CUST_57967_PI429347424	Carbonic anhydrase 12	CA12	*	*	-4.31	-7.23
**Unigene1622_TP**	CUST_1621_PI429347424	Chitin synthase	CHS	-19.52	-7.02	*	-5.58
**Unigene47913_TP**	CUST_4791_PI429347424	Calcineurin A	CALA	-5.08	-11.84	-3.04	-4.60
**Unigene50693_TP**	CUST_50654_PI429347424	Calmodulin	CAM	-2.49	-2.64	-4.45	-3.67
**Unigene31411_TP**	CUST_31390_PI429347424	Calmodulin-2	CAM2	*	-2.01	*	*
**Unigene29790_TP**	CUST_29769_PI429347424	Calmodulin-1b	CAM1B	*	-2.02	*	*

Supplementary Table 7. Parameters of seawater chemistry measured and calculated (CO_2SYS) for each experimental treatment (Mean ± SD). Abbreviations: TA: total alkalinity; DIC: dissolved inorganic carbon; pCO_2: partial pressure of CO_2; [HCO_3^-]: HCO_3^- concentration; [CO_3^{2-}]: CO_3^{2-} concentration; Ωca and Ωar: saturation states for calcite and aragonite.

Symbol	Measured parameters					Calculated parameters				
	**pH**	**Temperature (° C)**	**Salinity (psu)**	**TA (µM/kg)**	**DIC (µM/kg)**	**pCO_2 (µatm)**	**[HCO_3^-] (µM/kg)**	**[CO_3^{2-}] (µM/kg)**	**Ωca**	**Ωar**
**Control-0**	8.12 ± 0.03	19.33 ± 0.47	33.00 ± 0.82	2427.28 ± 47.91	2139.90 ± 24.71	341.34 ± 19.20	1918.74 ± 12.80	209.48 ± 17.77	5.07 ± 0.43	3.27 ± 0.28
**Control-24**	8.09 ± 0.04	19.33 ± 0.47	33.00 ± 0.82	2489.33 ± 39.27	2210.03 ± 35.22	382.00 ± 8.50	1991.00 ± 16.43	205.00 ± 28.10	4.87 ± 0.88	3.21 ± 0.15
**Control-48**	8.12 ± 0.03	19.33 ± 0.47	33.00 ± 0.82	2488.28 ± 47.91	2231.90 ± 24.71	415.34 ± 19.20	2025.74 ± 12.80	192.48 ± 17.77	4.99 ± 0.43	3.01 ± 0.28
**Control-72**	8.08 ± 0.02	19.33 ± 0.94	33.00 ± 0.47	2493.46 ± 44.21	2225.00 ± 10.54	394.00 ± 20.01	2001.00 ± 33.22	199.00 ± 13.26	4.68 ± 0.24	3.13 ± 0.22
**T25-24**	8.13 ± 0.02	24.67 ± 0.47	33.33 ± 0.47	2399.56 ± 44.72	2046.10 ± 41.47	320.77 ± 18.97	1782.55 ± 39.07	254.31 ± 8.36	6.20 ± 0.18	4.07 ± 0.12
**T25-48**	8.08 ± 0.01	25.67 ± 0.47	33.67 ± 0.47	2366.19 ± 43.29	2037.21 ± 44.61	364.90 ± 17.08	1790.33 ± 44.51	236.65 ± 6.47	5.77 ± 0.14	3.80 ± 0.10
**T25-72**	8.15 ± 0.05	25.33 ± 0.47	33.67 ± 0.47	2345.32 ± 43.89	1981.97 ± 12.52	302.09 ± 42.26	1714.85 ± 24.29	258.50 ± 26.85	6.29 ± 0.66	4.14 ± 0.43
**T31-24**	8.12 ± 0.06	30.67 ± 0.47	33.00 ± 0.00	2401.23 ± 42.29	1997.99 ± 2.16	331.74 ± 52.47	1699.66 ± 30.21	290.05 ± 30.54	7.19 ± 0.76	4.81 ± 0.50
**T31-48**	8.11 ± 0.06	30.33 ± 0.47	34.33 ± 0.47	2397.32 ± 50.83	1990.28 ± 84.22	334.33 ± 68.93	1692.19 ± 105.32	289.73 ± 25.40	7.09 ± 0.61	4.75 ± 0.41
**T31-72**	8.11 ± 0.06	30.33 ± 0.94	33.67 ± 0.47	2410.55 ± 50.35	2002.83 ± 80.89	340.37 ± 58.98	1701.94 ± 34.25	292.49 ± 35.69	7.21 ± 0.88	4.84 ± 0.59
**P78-24**	7.80 ± 0.03	18.33 ± 0.47	34.33 ± 0.94	2459.77 ± 51.54	2320.92 ± 38.90	802.53 ± 44.67	2177.35 ± 31.62	116.05 ± 8.68	2.78 ± 0.21	1.80 ± 0.14
**P78-48**	7.78 ± 0.01	18.33 ± 0.94	34.33 ± 0.47	2542.94 ± 65.47	2405.11 ± 69.50	884.11 ± 53.91	2257.61 ± 68.34	118.03 ± 2.76	2.83 ± 0.06	1.83 ± 0.04
**P78-72**	7.77 ± 0.07	17.67 ± 0.47	33.67 ± 0.94	2617.46 ± 111.54	2490.23 ± 124.24	953.89 ± 187.79	2343.33 ± 125.52	114.08 ± 15.71	2.75 ± 0.39	1.77 ± 0.25
**P75-24**	7.51 ± 0.05	18.33 ± 0.47	33.67 ± 0.47	2528.40 ± 52.94	2496.86 ± 69.38	1720.27 ± 250.45	2373.63 ± 66.06	64.02 ± 5.22	1.54 ± 0.13	1.00 ± 0.08
**P75-48**	7.46 ± 0.05	18.33 ± 0.94	33.33 ± 0.47	2611.15 ± 147.32	2595.63 ± 147.48	2049.83 ± 270.96	2466.65 ± 140.30	60.26 ± 7.63	1.46 ± 0.19	0.94 ± 0.12
**P75-72**	7.47 ± 0.08	18.33 ± 0.47	33.67 ± 0.47	2768.35 ± 225.85	2749.18 ± 214.97	2072.49 ± 338.81	2612.15 ± 203.95	65.70 ± 15.13	1.58 ± 0.36	1.02 ± 0.23

Supplementary figures

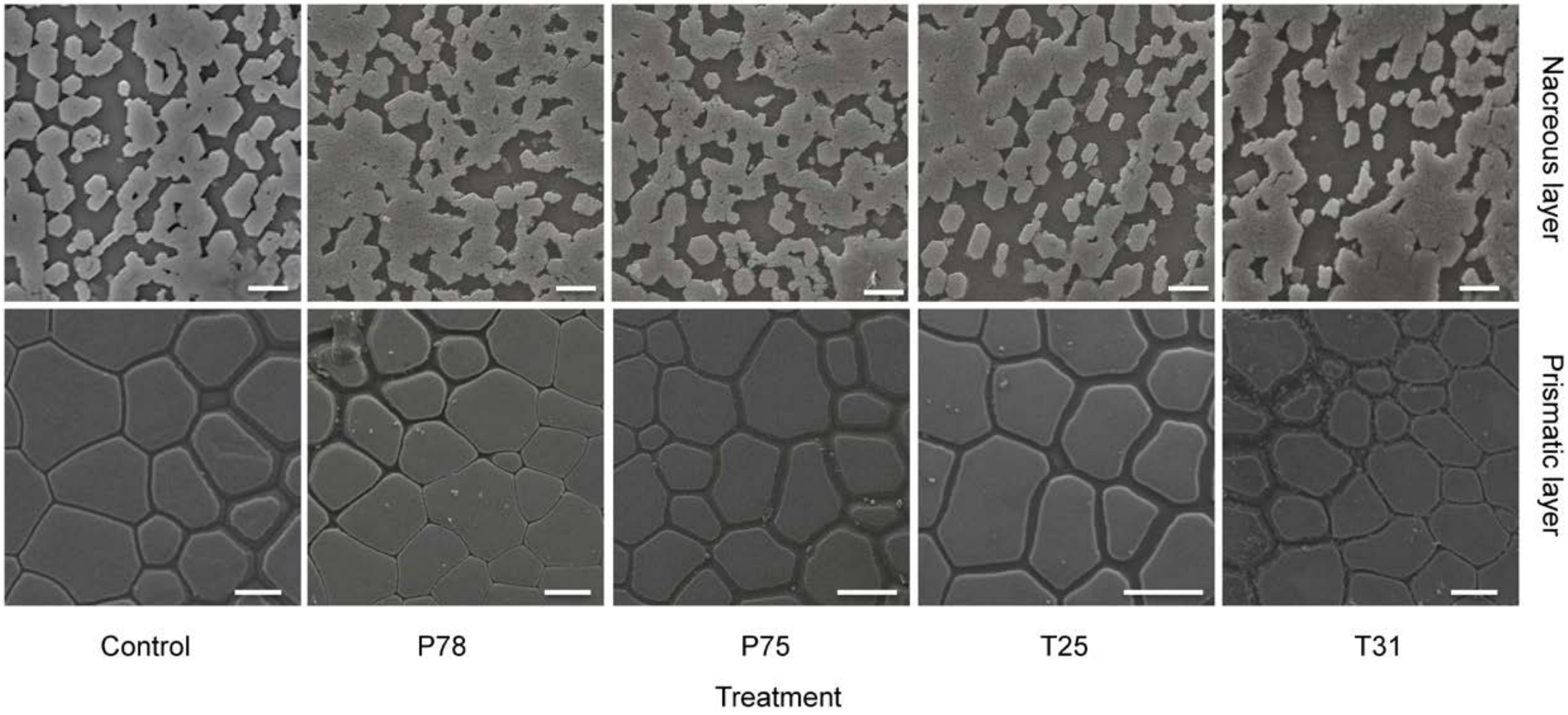

Supplementary Fig. 1. The scanning electron microscope (SEM) micrographs of the nacreous and prismatic layers in the shells of *Pinctada fucata* exposed to elevated CO_2 and temperature. Scale bar = 3 μm in nacreous layers. Scale bar = 25 μm in prismatic layers.

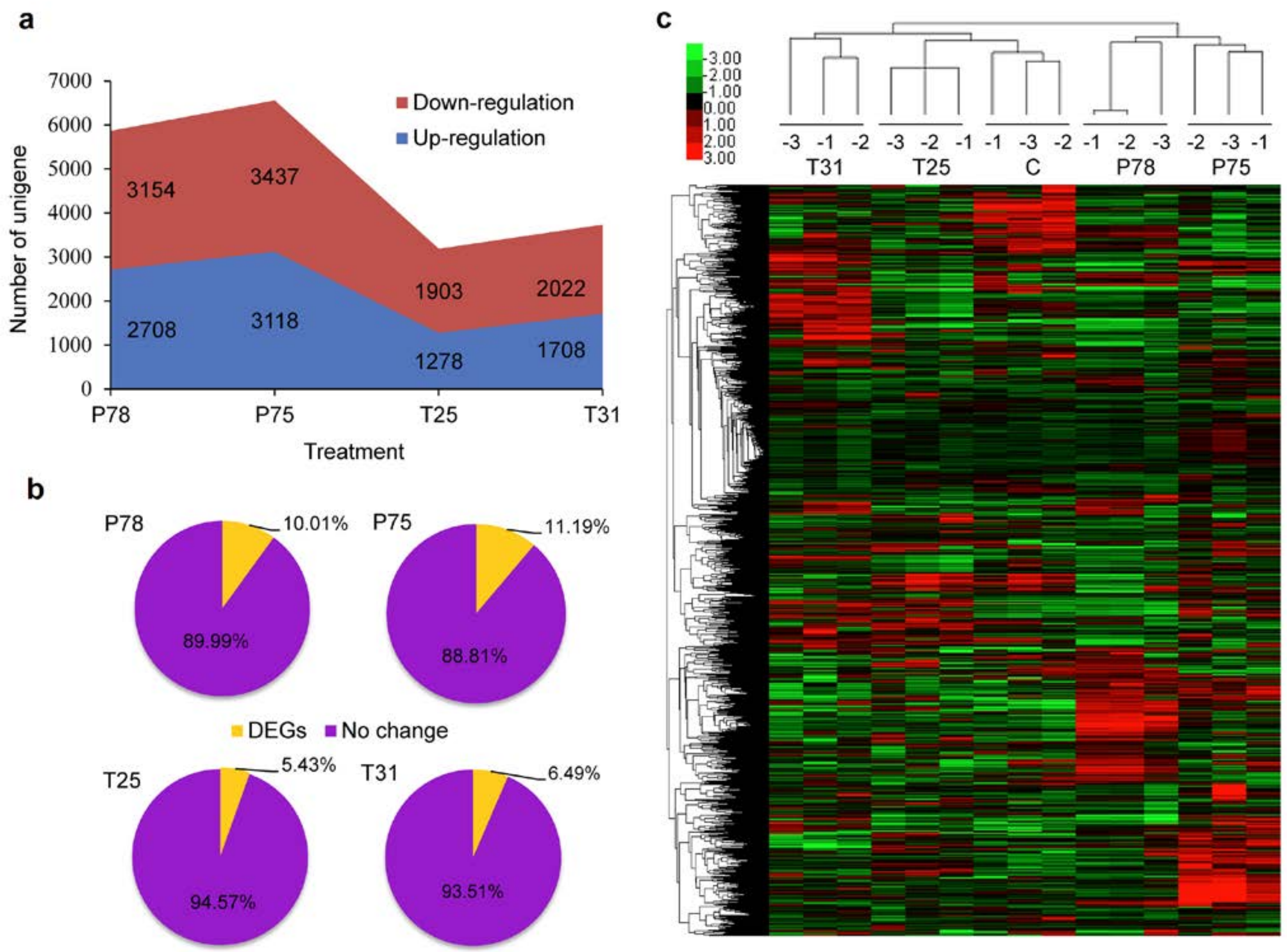

Supplementary Fig. 2. The numbers of up- and down-regulation (a), ratios (b) and heat map (c) of differentially expressed unigenes (DEGs) in *Pinctada fucata* exposed to elevated CO_2 and temperature. In the heat map, red indicates up-regulation; green indicates down-regulation and black indicates insignificant changes. The three biological replicates in each cluster are referred as -1, -2, and -3.

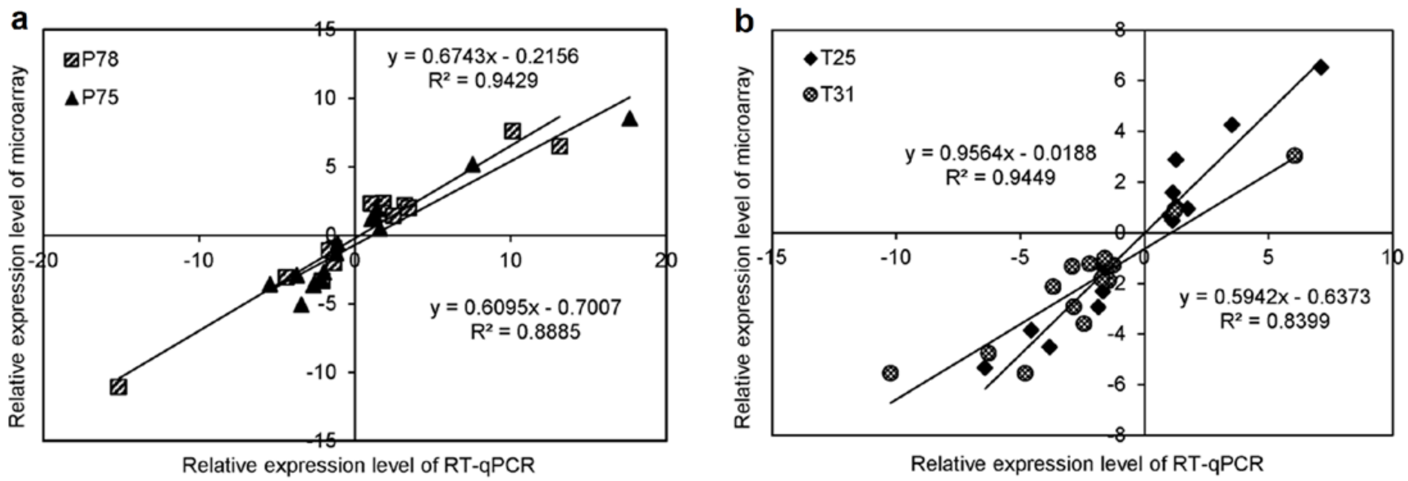

Supplementary Fig. 3. Correlation analysis between microarray and RT-qPCR results. Fourteen differentially expressed unigenes listed in Supplementary Table 2 were analyzed by RT-qPCR.

References:

1. Liang, J., Zhang, L., Xiang, Z. & He, N., Expression profile of cuticular genes of silkworm, *Bombyx mori*. *BMC Genomics* **11** 173 (2010).

2. Yano, M., Nagai, K., Morimoto, K. & Miyamoto, H., Shematrin: A family of glycine-rich structural proteins in the shell of the pearl oyster *Pinctada fucata*. *Comp. Biochem. Physiol. B Biochem. Mol. Biol.* **144** 254 (2006).

3. Livak, K. J. & Schmittgen, T. D., Analysis of relative gene expression data using real-time quantitative PCR and the $2^{-\Delta\Delta CT}$ method. *Methods* **25** 402 (2001).
